# Supplementary material for: Are carbon emissions trading and green financial instruments synergistic? -Comprehensive quantitative research based on content analysis
Source: PLoS One. 2024 Mar 7;19(3):e0298601. doi: 10.1371/journal.pone.0298601 (PMC10919601; doi:10.1371/journal.pone.0298601)
Supplement: S1 Data — (ZIP) [file pone.0298601.s001.zip › Data/地级市Z_F工作报告.zip!/╕╩╦α/░╫╥°╩╨/└┤╫╘╣1⁄2╓┌║┼í╛┬φ┐╦╩2╛▌═°í┐_2013.docx]

各位代表：

现在，我代表市人民政府，向大会作政府工作报告，请予审议，并请政协委员及列席人员提出意见。

关于2012年政府工作

今年以来，在省委、省政府和市委的正确领导下，市政府认真贯彻省第十二次党代会和市第七次党代会精神，牢牢把握稳中求进、好中求快的总基调， 积极应对经济运行中出现的新情况、新问题，经济社会保持了平稳较快发展的良好态势，预计年底可全面完成市八届人大一次会议确定的目标任务。

——经济运行再上新台阶。预计全年完成生产总值435亿元，同比增长14.1%；固定资产投资321亿元，增长45%；社会消费品零售总额 120.5亿元，增长18.2%；大口径财政收入52.47亿元，增长15.39%；公共财政预算收入23.31亿元，增长36.17%；公共财政预算支 出102.42亿元，增长21.33%；城镇居民人均可支配收入18560元，增长16.3%；农民人均纯收入4500元，增长18%；居民消费价格涨幅 控制在3%以内。

——项目建设取得新突破。全市在建项目714个，同比增加168个，其中亿元以上项目118个，同比增加45个；实施市列重点项目105个，完成投资79.5亿元。

——园区开发构筑新格局。白银工业集中区“一区六园”开发土地36.36平方公里，完成固定资产投资122.98亿元，其中基础设施投资17.97亿元；实现工业增加值86.65亿元，占全市工业增加值的56%。

——科技创新迈出新步伐。5万平方米科技孵化器一期工程建成投运，入驻企业26家；引进国家“百千万人才工程”第一层次人才1人、科研团队3个；新 增高新技术企业5家、创新型企业5家、企业技术中心3个、工程技术研究中心1个、产业技术创新战略联盟1个；“院士专家服务中心”挂牌成立。高新区世界首 座超导变电站被评为“中国十项重大科技工程进展”。

——循环经济渐成新亮点。实施循环经济项目79个，完成投资34.38亿元。高新区列入国家循环化改造示范试点园区，我市被评为“中国循环经济优秀品牌城市”。

今年以来，主要做了八个方面的工作：

一是抓规划、谋长远，生产力布局不断优化。开展“规划年”活动，确定编制规划64项，完成白银工业集中区、3个千亿元产业链、城市总体规划、防灾减 灾体系等专项规划42项，正在编制22项。配合编制兰西格经济区、六盘山片区区域发展与扶贫攻坚等规划，甘宁中部扶贫供水工程等18个重大项目纳入国家和 省级战略规划。

二是抓科技、调结构，农村经济稳步发展。创建高产万亩示范片20个，完成全膜双垄沟播127.45万亩、高效节水灌溉74.51万亩，引进新技术 33项，农作物良种覆盖率和畜禽良种普及率均达到95%以上。玉米、马铃薯面积稳定在100万亩以上；粮食总产量80.36万吨，增加9.96万吨，实现 “六连增”。新改建日光温室1万亩，新增瓜菜1.51万亩，枸杞、文冠果等特色林果16.23万亩。划定基本草原1838.97万亩，落实生态保护补助奖 励资金5214.81万元，承包到户25.5万户。新建规模养殖场76个，新增青贮饲料加工能力58万立方米，秸秆转化率达到55.32%，牛、羊饲养量 分别增长8.76%、4.46%；新建奶牛场8家，新增奶牛3000头。流转土地31.23万亩，新增龙头企业13个、农民专业合作社441个，获得国家 级农产品金奖5个、省级农产品金奖4个。争取扶贫专项资金1.3亿元，实施连片开发项目1个、整乡推进项目4个、整村推进项目22个。

三是抓项目、保增长，工业经济逆势攀升。在建工业项目286个，完成投资125.23亿元，同比增长36.94%。预计全年规模以上工业增加值 193亿元，增长17.3%。鸿泰铝业铝合金、东方钛业钛白粉等产业链项目开工建设；大唐、华电、中电投等新能源项目启动实施；白银有色集团铜冶炼、长通 电线电缆铜铝导体新型材料等技术改造提升项目开工建设；银光集团7.5万吨TDI、靖煤集团异地开发、寿鹿山水泥日产4500吨干法生产线等扩能改造项目 进展顺利；刘化集团年产30万吨硝酸等循环经济项目加紧建设；稀土公司高性能抛光粉、雄信铝业铝型材深加工、颐通管业PE实壁管等新材料项目建成投产；白 银有色集团重金属废水治理、银光集团废水深度处理等环保项目建成投运；甘藏银晨公司16万吨铬渣解毒、东大沟流域农田重金属污染治理示范工程基本完成；西 北矿冶院复杂多金属矿绿色高效捕收剂清洁生产与选矿新技术开发项目通过国家验收。期盼多年的热电联产项目今天奠基开工。预计全年万元生产总值能耗下降 8%，万元工业增加值能耗、用水量分别下降13%、5.9%。

四是抓基础、拓空间，承载能力不断增强。开展“五城联创”活动，完成白银城区大环境绿化7000亩，金岭公园、银光公园、银凤湖公园加快建设;新建 改造城区道路15条，亚行贷款一期城市道路、国道109线过境段改造等项目加快实施；改造市区供热供水管网52公里、交通通讯设施50余处，完成白银有色 集团供热、供水系统移交；建成全省首家城市地下管网探测管理、地上建筑物三维建模系统。推进白银工业集中区扩区增容，开发低丘缓坡荒滩等国有未利用土地 3.44万亩，高新区预计完成固定资产投资30.2亿元，增长50%，16平方公里核心区基础设施基本建成；银东工业园平整土地5000亩；银西工业园平 整土地1万亩；刘川工业园11条主次干道、10平方公里土地平整和生活服务区全面开工建设；平川工业园完成基础设施投资1.13亿元；会宁工业园平整土地 1600亩，开工基础设施项目5个，完成投资1.5亿元；正路工业园完成7公里路基工程。加快水利、交通等基础设施建设，双永供水、工农渠泵站改造工程进 展顺利，靖会工程甘沟干渠改扩建、兴电灌区齐家大岘改扩建工程年内开工建设；营盘水至双塔高速公路景泰段完成投资9.8亿元，沿黄快速通道白桦连接线开工 建设，新建农村公路400公里、通乡油路80公里。

五是抓融资、促招商，开放开发进程加快。全市发放龙头企业、“公司+农户”等涉农贷款112.74亿元，增长20.49%；重点项目中长期固定资产 贷款55.65亿元，增长18.35%；中小企业贷款29.5亿元；累计发放城镇失业人员、妇女小额担保贷款27.16亿元；新设立小额担保贷款公司6 家。市财政设立生物科技产业基础资金1000万元，安排重点用能企业补助资金260.64万元、中小企业扶持资金2000万元。靖煤集团上市已获证监会批 准；菁茂农业科技股份公司在天津股权交易所挂牌交易，为我省首例。市国有资产开发经营公司、水务集团成功组建，三县成立政府投资担保机构，13户企业通过 “银政投”融资1.86亿元。签署兰州——白银区域合作发展行动计划，与福建泉州、安徽芜湖结为友好城市，实现与兰州、上海等七个城市公交IC卡互联互 通。实行全员招商，安排项目前期经费5000多万元，突出产业链点对点招商，制定考核奖惩办法，开展帮办服务，先后引进青岛德正等一批先进高载能企业和产 业链项目；“兰洽会”签约项目156个，签约资金849亿元；合同项目133个，签约资金368亿元；落地开工85个，续建和新开工项目完成投资75亿 元，增长25%。

六是抓商贸、兴三产，文化旅游加快发展。改造完成“万村千乡”市场工程农家店200家，新建县乡商品配送中心9家、放心粮油示范店20家。开通市家 政服务网络平台，扶持家政服务龙头企业4家、加盟企业400多家。文化出版和广播影视部门整体合并，市县电视网络公司成立并完成移交，靖远、会宁两县秦剧 团完成改制，市群艺馆、博物馆、图书馆、渡河战役纪念馆等文化教育基地免费开放；新建农家书屋37个、乡镇综合文化站60个、村级文化大院10个，广播电 视“村村通”完成3.8万户、“户户通”完成19万户。黄河大峡谷旅游开发公司、会宁西雁传媒公司等一批文化企业相继组建；尚文坊文化商业街、新天地文化 商业街二期、长征胜利景园二期等产业基地建成运营，黄河假日城、大地院线影院等产业项目启动建设，预计全年文化产业完成增加值5.8亿元，增长35%。第 四届金凤凰少儿艺术大赛、环青海湖国际公路自行车赛白银段、全国男子自由式摔跤锦标赛等重大赛事在白银成功举办；爱乐合唱团荣获第十一届中国国际合唱节银 奖。建成靖远嘏萃园等旅游休闲度假基地，新增“农家乐”71户，加大黄河石林等品牌景区宣传力度，黄河风情文化节、会宁民俗文化节等旅游节会影响不断扩 大，全市接待游客342.75万人次，旅游收入18.12亿元，分别增长43%、44%。

七是抓和谐、惠民生，人民生活水平显著提高。优化支出结构，着力保障和改善民生，预计全年市财政用于教育、卫生、社会保障和就业支出46.68亿 元，比上年增加8.2亿元。深入开展全民创业工程，发放就业创业补贴1.2亿元，全年新增城镇就业3.1万人，城镇登记失业率控制在4%以内；完成劳务输 转30万人，创劳务收入31亿元。城乡居民社会养老保险实现全覆盖，全市2.04万名“五七工”、“家属工”全部纳入企业基本养老保险。城乡低保标准分别 提高15.5%、35.8%，农村五保供养标准提高400元。保障性安居工程年内可竣工1.41万套，改造农村危旧房1.53万户；12.83万农村人口 饮水安全问题得到解决。白银矿冶职业技术学院新校区开工建设；新改扩建校舍23万平方米、幼儿园31所，建成职业教育实训基地6个；农村义务教育学生营养 改善计划全面实施，受益学生13.31万人。省中医院托管共建市中西医结合医院，制剂中心开工建设；各县区人民医院住院部和门诊大楼投入使用，白银有色集 团、银光集团职工医院整体移交，县、乡、村公办医疗机构实现基本药物零差率销售全覆盖；新农合人均筹资标准由230元提高到290元。计生“五大工程”稳 步推进，全年人口自然增长率5.85‰。15个防灾减灾项目全部完工，发放救助资金5.4亿元；帮扶援建的碌曲则岔石林游客服务中心建成投运。13件惠民 实事目前5件已落实，8件抓紧办理，年底可全部完成。我市第三次蝉联“全国双拥模范城”。

八是抓效能、转作风，政府公信力和执行力有效提升。全面开展“效能风暴”行动，建立大督查工作机制，加强纠风工作，开展民主评议，初步形成了争项 目、比效能、抓落实、干实事的良好氛围，县区和市直部门向上新争取资金70亿元以上。政府政务公开、依法行政工作有序推进，取消调整行政审批事项651 项，减幅达到70%；受理审批事项15.27万件，办结率99.4%；办理人大代表建议和政协委员提案311件；审查颁布规范性文件28件；市级各部门、 各单位基本实现公文网上流转。“六五”普法全面开展，社会管理创新、矛盾化解、公正廉洁执法工作不断加强。创新人才培养工程启动实施，选拔人才70人，在 卫生系统聘任首席专家10名。深入开展“两联两扶、为民富民”行动，落实项目资金1076万元、资助资金3226.5万元，协调贷款4.94亿元，兴办实 事3924件。

各位代表，回顾2012年工作，我们深感成绩来之不易，在严峻复杂的经济形势面前，全市人民迎难而上，奋力拼搏，在攻坚克难中保持稳定增长，在苦干 实干中推进转型发展。我们深切地体会到，省委省政府和市委的坚强领导、果断决策，是我们做好一切工作的根本保证；全市人民同心同德、锐意进取，是我们战胜 困难、加快发展的力量源泉，这其中也凝结着各位代表、各位委员的智慧和力量，饱含着各级干部的心血和汗水。在此，我代表市人民政府，向全市人民，向各民主 党派、人民团体，向驻银部队、武警官兵，向中央、省驻银各单位，向所有关心、支持白银转型发展的各界人士，致以崇高的敬意和衷心的感谢！

在肯定成绩的同时，我们也清醒地认识到，经济社会发展还面临一些突出矛盾和问题，主要表现在：一是经济总量还不够大，三次产业发展不协调，第三产业 和建筑业增长缓慢，调整经济结构、转变发展方式、推动优化升级任重而道远；二是固定资产投资增速虽然为近年来最快，但带动产业升级的大项目偏少，同时受土 地、融资等因素影响，个别项目进展不快；三是城乡建设欠账较多，区域发展不平衡，农村交通、人居、生态环境等还不尽如人意，村容村貌还不够整洁，居民总体 收入水平还不高，与经济发展的态势不相协调；四是支撑科学发展的体制机制还不够健全，中小企业贷款融资难问题依然存在，安全生产责任落实还不到位，政府工 作与跨越发展的要求和人民群众的期望还有不小差距。对此，我们必须高度重视，认真加以解决。

2013年的目标任务和重点工作

2013年，是全面贯彻党的十八大精神的第一年。以十八大召开为标志，我国将开启改革开放和现代化建设新征程，推进科学发展、增进民生福祉、 促进社会和谐的举措更加有力，我们将迎来新一轮的大开发大发展。从政策环境看，国家“大力支持兰白核心经济区率先发展，建设兰白都市经济圈，积极推进兰州 新区、白银工业集中区发展”，省委省政府确定的区域发展战略等一系列为白银量身定做的特殊扶持政策，为我们更好地担当“三大责任”、集聚外部资源、加速跨 越发展提供了难得的历史机遇；从产业方向看，国际金融危机催生新的科技和产业革命，新能源、新材料、信息技术、绿色经济、生物经济发展方兴未艾，西部与东 部处在同一起跑线上，为我们大力发展新兴产业、缩小差距创造了有利条件；从发展基础看，经过近年来的不懈努力、真抓实干，我们已经奠定了厚积薄发、加速赶 超的良好基础，随着白银工业集中区开发建设步伐加快，我们必将进入一个空间潜力充分拓展、各种优势竞相迸发的全新时期。同时也要看到，受国际金融危机影 响，世界经济复苏乏力，我国经济发展的不确定性和不可预见因素增多。我们务必认清形势、正视困难，抢抓机遇、锐意进取，更加奋发有为地推进白银经济社会又 好又快发展，加快与全国同步进入全面小康社会的进程；务必增强责任意识和忧患意识，负重前行，开拓创新，努力实现经济社会发展水平的大幅提升，让全市人民 生活得更加富裕、更加和谐、更加幸福。

政府工作的总体要求是：认真学习贯彻党的十八大精神，以科学发展为主题，以加快转变经济发展方式为主线，以显著增加城乡居民收入为目标，坚持稳中求 进的总基调，加快结构调整和产业升级，加快白银工业集中区开发建设，加快基础设施改造提升，加快工业化、信息化、城镇化和农业现代化进程，创新发展、循环 发展、集聚发展、和谐发展，保持经济社会又好又快发展的良好势头，为与全国同步进入全面小康社会奠定坚实基础。

全市经济社会发展主要预期目标是：生产总值增长14%，工业增加值增长17%，公共财政预算收入增长15%，固定资产投资增长40%，社会消费品零 售总额增长18%，城镇居民人均可支配收入增长15%，农民人均纯收入增长17%，城镇登记失业率控制在4%以内，人口自然增长率控制在7.3‰以内，万 元生产总值能耗和污染物排放总量完成省上下达的控制指标。重点抓好以下十一个方面的工作：

（一）坚持重点突破，不断壮大区域经济总量。把兰白核心经济区、白银工业集中区作为转型跨越的主阵地，一手抓基础建设，一手抓产业集聚，推进兰白融合发展，构建“一区六园”板块经济，形成新的区域经济增长极。

共建兰白核心经济区。抓住兰州新区进入国家级新区战略机遇，坚持规划编制一体化、生产力布局统筹化、基础设施同城化，主动对接、优势互补、错位发 展、同步迈进。结合兰西格经济区发展规划编制，做好与兰州新区规划对接，把我市重大产业、重大项目纳入总体规划；合理布局产业项目，与兰州市联手争取黄河 上游兰白段生态修复及环境综合治理示范区、国家级承接产业转移示范区，共同建设兰白高速和沿黄经济带、国家新型工业化产业基地；加快推进轨道交通、高速公 路、高等级公路等重大交通基础设施建设，协作打造兰白黄河沿线特色城镇群，尽快实现两市通信融合，增强同城效应。

推进白银工业集中区建设。积极申请国家比照兰州新区给予白银工业集中区优惠政策，争取省政府出台支持白银工业集中区加快发展的意见。创新园区运行机 制，建立健全考核评价体系，加大投入力度，园区财税收入市县留存部分全部用于园区滚动发展。分类推进园区建设，高新区围绕3年内建设成为国家级科技孵化器 目标，强化孵化创新功能，重点发展生物医药、新材料、先进装备制造等高新技术产业，孵化企业20家，高新技术产业增加值增长20%以上；银东工业园启动建 设循环化工园、再生资源回收利用园和仓储物流园，重点发展精细化工、资源循环利用、生产性服务业；银西工业园完成三期土地平整2800亩，建成道路20公 里，实施集中供热一期工程，工业增加值增长80%；刘川工业园建成主次干道11条，完成23平方公里土地平整和给排水主网等基础设施配套工程，启动建设行 政商贸生活服务区，加快建设稀土新材料园，工业增加值增长26%；平川工业园重点推进南区开发，完成基础设施投资1.3亿元，工业增加值增长20%；会宁 工业园重点发展服装、生态食品、电器加工等劳动密集型产业，完成投资5.2亿元，工业增加值增长26%；正路工业园重点实施260万方调蓄水库、6公里道 路、110KV变电站及综合服务中心等项目，主动对接和服务兰州新区。

（二）壮大产业集群，构筑现代产业格局。实施“新型工业化发展计划”，启动建设3个千亿元产业链、4个百亿元产业，构建“3+4”产业集群，规模以上工业增加值增长17%。

提升特色优势产业。用高新技术改造有色、化工、能源首位产业。做强有色产业，加快实施白银有色集团铜锌冶炼技术提升、铜矿资源、铅锌资源开发项目，稀土公 司稀土资源开发项目，建设有色金属特色产业基地。做精化工产业，推进银光公司TDI扩建、PC新建、刘化集团硝基复合肥二期、重庆昌元高锰酸钾、东方钛业 钛白粉等项目建设，延伸化工产业链条。壮大能源产业，带动高载能产业，开工建设靖南峡水电站、垃圾焚烧发电、景泰和靖远山地风电、中电国际、中电投等光伏 发电项目，加快热电联产、寿鹿山水泥配套9兆瓦纯低温余热发电项目建设进度；重点实施鸿泰铝业百万吨铝合金产业链项目，增强电力消纳能力。

培育战略性新兴产业。大力发展新材料产业，加快实施甘肃石火科技锂电池及正极材料、郝氏碳纤维复合材料制品等项目。培育壮大生物医药产业，实施生物 科技加速器、生物医药科技产业基地项目，加快杰康诺微生物研发中心建设，支持平川赛诺、熙瑞，会宁长征药业等企业开发天然药物、功能饮料、保健养生等系列 产品，打造总面积20平方公里的生物经济园区。着力抓好资源循环利用产业，启动西北资源回收循环利用示范园建设，实施长盛废旧汽车回收拆解利用等项目，建 设再生资源交易中心。积极扶持高端装备制造产业，实施甘肃倚银石化装备制造公司过程分析设备生产基地、甘肃容和矿用设备技术改造、北京亦庄风电装备制造等 项目。

扶持富民多元产业。围绕三县进入全省县域经济20强的目标，争取扩权强县试点，加快县城扩容提质，促进劳动密集型产业和农产品加工业向县城和中心城 镇集聚；发挥两区工业主导优势，扩大城市规模，推进户籍、公共服务和社会保障综合配套改革，带动城乡一体化。全面落实各项就业政策，深入开展全民创业行 动，加快创业项目库和创业孵化基地建设，完成职业技能培训4万人、创业培训3000人，新增就业2.9万人；输转劳动力30万人，实现劳务收入30亿元以 上。认真落实国务院非公经济“新36条”及42项实施细则，出台推进民间投资的政策措施，举办民间投资项目推介会。实施“小微企业成长计划”，加快县区中 小企业产业园建设，培育“小巨人”企业5-10户，新增小微企业300户。

（三）积极培育现代农业，稳步推进农业农村经济发展。把“三农”工作作为转型跨越的重中之重，坚持工业化理念、市场化运作，推动农业向产业化、规模化、标准化、品牌化转变，促进农业增效、农民增收。

夯实现代农业发展基础。全市粮播面积达到350万亩，粮食总产量稳定在78万吨以上。建设国家级旱作农业示范区，推广全膜双垄沟播种植128万亩、 全膜覆土穴播冬小麦8万亩；建设马铃薯种薯等繁育基地96.7万亩，杂交玉米繁育基地6.8万亩，创建高产万亩示范片10个。推广先进适用农机技术装备， 投放青贮饲料加工成套设备300套，补贴农机具购置资金3000万元，农机总动力达到215万千瓦，农业机械化综合作业水平达到40%。

打造现代农业产业体系。调整内部结构，新改建日光温室1万亩、塑料大棚6000亩；新增大枣、枸杞、核桃等特色林果业和林下经济10万亩；新增经济 作物2万亩；新建标准化规模养殖场50个，奶牛场5家，新增奶牛5000头；新发展龙头企业10家，农产品加工转化率40%以上。强化科技支撑，发展高效 农田节水灌溉87.7万亩、测土配方施肥360万亩；引进新技术40项、新品种280个，良种覆盖率保持95%以上；推进靖远大坝、白银区重坪农业园区晋 级省级示范园区，创建会宁现代草产业科技园。培育特色品牌，建设国家级农作物品种区域试验站、会宁和靖远农业有害生物预警站，健全农产品质量检测体系；创 建无公害农产品基地5个，争取获得省级以上农产品金奖5个，扩大“三品一标”农产品覆盖面，在靖远县、白银区推行蔬菜产地准出和市场准入制。

完善现代农业经营制度。推进集体林权制度综合配套改革，促进林地林权流转。建立健全三级土地流转市场服务体系，争取景泰县列入省级土地流转服务中心 建设示范县；全市扶持土地流转经营大户50户，土地流转40万亩。新增农民专业合作社210家，加入合作社的农户比重提高3个百分点，50%以上的农产品 通过合作社加工、销售。

（四）大力发展现代服务业，推动第三产业提质增速。把加快发展服务业作为转型跨越的有效途径，全面落实税收、土地等优惠政策，确保服务业增加值增速达到14%。

加快发展生产性服务业。围绕服务白银工业集中区开发建设，发展现代物流业，完善商贸物流体系规划，建设银西中进现代仓储物流园、白银有色集团工业物 流园、靖远润辰汽车物流园、平川能源建材物流园和白银品牌汽车市场、正路工业园建材市场，发展一体配送、货运代理、电子商务、直购直销等新型业态。支持国 泰君安证券、兰州银行、招商银行、浦发银行等在我市设立分支机构，继续深化农村信用社改革，规范发展小额贷款公司等地方小微金融组织。开工建设商会大厦。 大力发展法律、会计、咨询、信息、设计等面向生产的服务业。

大力发展生活性服务业。建设环银百大楼、环西区人民广场、环文化广场三个商业中心，抓好义乌小商品市场、兆祥商贸中心、平川春茂城市广场、新疆好好 成食品生猪屠宰及肉品加工、万吨冷链物流等项目建设。加快农产品批发市场改造升级，重点打造白银粮油、靖远北方菜业、白银大井子农产品等专业批发市场，规 划建设工农路星级酒店，推动住宿、餐饮、交通运输等传统服务业转型升级。开展医疗卫生、家政、文化娱乐、职业指导等便民服务，创建全国家政服务体系试点城 市。

（五）实施项目带动行动，确保固定资产投资较快增长。把项目建设作为转型跨越的着力点，强力推进全省“3341”项目工程，以项目拉动投资、以项目 调优结构、以项目改善民生，实施项目601个，固定资产投资增长40%，其中市列重点项目109个，完成投资183亿元。

千方百计谋划项目。深度解读国家宏观政策、产业导向和投资需求，建立近远期结合、覆盖经济社会各行业的项目储备库。实行项目入库备案制、定期申报 制、定期调度制，及时补充扩容项目库，谋划一批投资规模大、市场前景好、产业关联度高、带动能力强的好项目、大项目，为全省项目观摩“看大不看小”打好基 础、做好准备。

加大力度建设项目。坚持大中小项目协调推进，突出抓好大体量项目，实施亿元以上项目146个，10亿元以上项目11个。对计划开工项目，组成专门工 作班子，“一对一”跟进，明确时间节点，创造条件开工建设；对在建项目，倒排工期，加快进度，确保完成年度投资计划；对已经运营的项目，定人定点、一企一 策，确保达产达标。

全员招商引进项目。建立长三角、珠三角、环渤海等重点区域和中国500强等重点企业数据库，围绕3个千亿元产业链，找准缺失环节，补链招商，精准招 商，配套上下游企业。按照共建共享、收益分成模式，在园区设立“飞地经济”区，吸引企业和投资集团整块开发。坚持全员招商，完善激励奖惩机制，提高项目履 约率、资金到位率、开工投产率；引进合同项目150个，项目履约率70%以上，投资增长30%以上。

全力以赴保障项目。完善建设用地审查报批程序，健全重大项目用地事前协商制度，新增用地优先用于重大产业项目；依法依规限制企业在土地、能源、矿产开发中 圈占、闲置、擅自改变用途等行为。建立企业资金需求目录，制定项目融资方案，强化“银政园企”合作，扩大“银政投”授信额度。实行每个重大项目一名市级领 导挂帅、部门一把手主抓、一个明确的时限、一套推进措施制度，严格执行周报月报、跟踪督查和例会制，定期召开项目办公会、调度会和协调会，推行“管家 式”、“帮办式”服务，确保项目顺利推进。

（六）坚持基础优先取向，增强经济发展后劲。把夯实基础作为转型跨越的重要保障，推进交通、水利、信息、生态项目建设，破解发展瓶颈制约。

完善交通网络。抢抓国家支持西部铁路建设机遇，重点抓好包兰线红会支线扩能改造延伸至环县、白银至平凉铁路、干塘至武威南增建二线白银段、包兰线银川至兰 州扩能白银段等项目前期工作。建设景泰至兰州高速公路、白银至中川机场二级公路、兰州至黄河石林二级公路、白银至景泰大水[砣]　　公路、省道207线靖 远至会宁侯川段改建等工程,确保营双高速公路景泰段、坝滩至吴家川公路建成通车,新建高速公路62公里、农村公路400公里、通乡油路80公里;推进农村 公路养管体制改革,县乡公路好路率达到74.38%。开工建设乌金峡库区及龙湾至南长滩航运项目。

加强水利建设。加快引洮一期会宁北部供水、双永供水、靖会工程甘沟干渠改扩建、兴电灌区齐家大岘改扩建工程建设，推进引洮二期、甘宁中部生态移民扶 贫开发供水工程、黑山峡梯级开发前期工作。实施中小河流治理项目5个，争取实施河西走廊国家级高效节水灌溉、黄河干流白银段防洪治理项目。建设农村安全饮 水工程20处，解决15万以上农村人口饮水安全问题。新增有效灌溉面积2.5万亩，新修梯田15万亩，水土保持综合治理150平方公里。

加快信息化步伐。推动“三网”融合，建设“数字城市”。加强电子政务、应急管理网络平台建设，推进食品安全、市场管理、智能交通、应急处理突发事件 等数字平台建设，推动电子政务向乡镇、街道、社区延伸，向教育、卫生、文化等公共服务发展，建设“三维数字社会管理”系统，打造“智慧白银”。

（七）加快新型城镇化进程，建设生态宜居城市。把推进城镇化作为转型跨越的重要抓手,以城镇基础建设为重点,构建功能完善、布局优化、运转有序的宜居宜业城市。

科学编制规划。实施白银旧城区、市政工程、水系和城市环境色彩等规划，完成银东工业园、银西工业园、白银有色集团工业片区、银光集团生活区、平川城 区等规划编制，做好小城镇和乡村规划编制，村庄规划覆盖率达到100％。强化规划监督管理，维护规划的连续性、权威性、严肃性。

完	善城市功能。实施城市建设“44489工程”，完成银西、银东生态湿地建设，启动白银区政府原办公楼等4个片区旧城改造工程，大井子等4个片区城 中村改造工程，新建兰包路东入口广场等4个街区广场，改造完成109国道城区过境东段、人民路等8条道路，新建北环路、银西中路等9条道路。完善供水、供 暖、供气、排水、污水处理、垃圾处理等基础设施，整合供热企业，新建北城区热源厂和2个出租车加气站。开工建设银凤湖五星级大酒店、仿古一条街、银西片区 中央商务区等标志性工程。规划建设白银东城区公交枢纽站。完成各类保障性住房14070套；改造农村危房1万户、8万平方米。实施银凤湖公园、银光公园二 期、矿山公园、西山公园、西大沟延伸治理、平川沁川湖等城市绿化工程，建设小游园、小绿地、小街景，完成白银城区大环境绿化5000亩。

加强城市管理。结合“五城联创”，开展城市示范性街道、门头牌匾、户外广告、色彩集中整治行动，完善城区道路交通信号灯和标志标线，加强道路两边 “店外店”划行归市管理。严格落实“门前四包”、垃圾清扫保洁、日产日清等制度。加大餐厨垃圾无害化处理力度，统筹规划生活、医疗和建筑垃圾处理场站建 设。

注重经营城市。树立“以城养城、以城建城”的现代城市经营理念，建立投资多元化、运营市场化的城市经营机制。推行特许经营制度，引入BT、B0T等 项目融资模式兴建基础设施。争取国家开发银行政策性贷款，利用好世界银行和各国政府低息贷款，完成亚行二期城市贷款项目前期工作。充分发挥市城投公司的作 用，注入经营性资产，整合优质资产，盘活有形资产，强化土地储备，多渠道筹措城市建设资金。对户外广告标牌、公共客运线路、道路冠名权等无形资产，通过转 让、拍卖、租赁、置换等有效途径，转化成有形项目，吸纳社会资金参与城市建设。

推进特色城镇化。统筹城乡发展，推进城乡发展规划、产业布局、基础设施、资源配置、公共服务、就业保障、生态建设、社会管理等“八个一体化”，引导 农民向城镇集中、土地向规模经营集中、公共服务向新型社区集中，城镇化率提高1.5个百分点。以交通干线为骨架，以城市周边、公路沿线、黄河沿线、旅游景 区为重点，科学确定村镇体系；借助实施兰白沿黄快速通道项目，谋划建设沿黄小城镇群；用好农村重点小城镇建设资金，采取以奖代补、贷款贴息方式，重点支持 郭城、刘川、红水、水川、四龙、水泉6个乡镇，打造全市小城镇建设示范镇。结合国家路网建设，打通城镇对外联系通道，把基础设施规划建设重点向城乡结合 部、重点镇延伸，逐步实现城乡公交化。突出城镇产业功能，坚持“一镇一业”，统筹制定中长期产业发展规划，确定重点发展产业，推进产业体系建设。强化小城 镇的纽带作用，促进农业、农村资源向工业和城镇转移。

加快新农村建设。强力推进农村道路、水利、电力等基础设施建设，整合项目，加大投入，市县联动，企业扶持，加快建设农村新型社区，抓好河畔、太平 店、乌兰、五合、喜泉、草窝滩、共和7个县级示范镇建设，指导村民建设经济安全适用、节地节能节材的新农村住宅，着力解决“有新房无新村”的现象；加大农 村环境整治力度，全面推进畜禽养殖污染治理、农村生活污水、生活垃圾治理等综合整治工程，改善农村生态环境。突出主导产业，大力发展村级集体经济，促进产 业发展与新农村建设互动相融，不断缩小城乡发展差距。

（八）突出创新引领，努力建设创新型城市。把科技创新作为转型跨越的强大动力，启动创新型城市建设，健全区域创新体系，推动经济发展方式由要素驱动向创新驱动转变，再创全国科技进步先进市。

强化企业创新主体地位。建立以企业为主体的技术创新体系，加大技术研发、专利申请资助等专项经费投入，利用无息借款、贷款贴息、贷款担保等方式，推 广创新成果。支持大型企业建立研发机构，支持中小企业向科技型和高新技术企业发展；加强关键技术攻关，做好引进技术的消化吸收再创新，新增创新型企业5 家、科技型企业30家、高新技术企业3家。建设国家知识产权试点城市，新增全国知识产权试点单位3家，专利申请量增长30%，万人发明专利拥有量达到 0.5件。

搭建科技成果转化平台。建设孵化器二期工程，发挥科技孵化器公司作用，引导社会资金参与，争取10个以上项目获得国家和省上科技型中小企业创新基金 支持，发展孵化经济。加快重点企业创新载体建设，建成省市级工程技术研究中心3个以上、企业技术中心3个。建立健全以产学研合作中心、创业服务中心为主体 的科技中介服务机构。发展创投、风投、信用担保机构，吸引科技企业设立分支机构，发展科技投资业务。

加强创新人才队伍建设。实施“铜城学者”和产业创新团队计划，设立企业院士专家工作站和博士后工作站。围绕高新区和企业工程技术中心，引进行业领军 人才和知名专家。加快“人才特区”建设，加大创新人才培养力度，支持企业对有特殊贡献人才实行股权奖励、技术入股等股权分配激励机制，落实各类优惠政策， 建立高层次人才岗位补贴和保障制度。

（九）注重循环发展，加快建设“两型”社会。把“两型”社会建设作为转型跨越的方向，倡导节约、环保、文明的生产方式和消费模式，让节约资源、保护环境成为全社会的自觉行动。

加快循环经济示范区建设。建设有色金属（稀土）深加工及新材料、化工、能源和先进高载能3个千亿元循环经济产业链。实施白银有色集团堆存铜冶炼弃 渣、扎布耶锂业碳酸锂深加工苛化废渣、正生生物废弃物、宏发电力电石渣等综合利用项目，大力发展矿产资源、固体废物、再生资源、餐厨废弃物等资源循环利用 产业，创建国家循环经济示范城市、国家废旧商品回收体系示范城市和国家“城市矿产”示范基地。发展农牧复合型生态循环农业,建设农村清洁示范工程2处，秸 秆利用率58%以上，废旧农膜、尾菜回收利用率均达到60%以上。

加强节能减排。严格落实节能减排目标责任制，抓好三县一区污水处理厂建设，实施大唐景泰电厂和靖远二电公司脱硫、脱硝工程，建设甘肃节能减排示范城 市。强化对重点领域、重点企业和重点污染源预警监管，严防“十五小”和“新五小”企业死灰复燃。注重建筑节能和公共机构节能，确保完成节能减排目标任务。

推进生态环境建设。继续落实草原生态保护补助奖励政策，抓好天然林保护二期、退耕还林、三北防护林等重点生态工程，新增造林面积32万亩。争取东大 沟流域重金属污染整治及生态修复项目列入西部生态规划和大江大河治理规划。推进低丘缓坡荒滩等未利用地开发，削山造地2万亩。实施双永工程土地开发项目， 整理土地1万亩。

（十）挖掘特色资源，促进文化旅游繁荣发展。把打造先进文化作为转型跨越的重要源泉，推进文化与旅游的融合发展。

完善公共文化服务。抓好市文化艺术中心、社区文化中心和村文化活动室建设，完善数字图书馆、公共电子阅览室等公共文化设施。建成无线发射台站、移动多媒体优化覆盖、广播电视“户户通”后续数字地面无线覆盖工程。

培育文化产业。围绕建设甘肃华夏文明保护传承与创新发展示范区，抓好红色文化产业园、黄河文化产业园、陶瓷文化产业园、工矿遗址实景体验基地等12个文化产业基地建设。认定一批市级文化产业重点企业，扶持壮大黄河大峡谷文化旅游、陇烨艺术陶瓷集团等文化企业。

提升旅游产业。突出黄河石林品牌效应，建设5A级景区；实施全国红色旅游名城和国家历史文化名城创建工程，扩大会师圣地影响力；开工建设永泰龟城修 复保护和开发项目，保护五佛寺等丝路石窟群和长城遗址。建成黄河假日城一期工程，开工建设二期工程，建设龙湾古镇、靖远鱼龙山等文化旅游体验景区，完善 “吃住行游购娱”旅游链条，发展“农家乐”示范村5个、示范户20户以上。举办全国黄河文化旅游博览会。旅游接待人数突破400万人次，旅游综合收入突破 20亿元。

（十一）坚持共建共享，不断增进人民福祉。把社会和谐作为转型跨越的最终目的，解决关系群众切身利益的生活、生产和生命安全问题，大幅提升人民群众幸福指数。

加大扶贫开发力度。落实各项强农惠农政策，深入开展“两联两扶、为民富民”行动，巩固专项扶贫、行业扶贫和社会扶贫“三位一体”扶贫格局；实施片区 开发及整乡整村推进项目20个，完成易地扶贫搬迁508户2417人，减少贫困人口10%，扶贫对象人均纯收入增幅高于全市平均水平2个百分点。

发展公共公益事业。推进义务教育阶段学校标准化建设，新改扩建校舍15万平方米，消除危房10万平方米，改造薄弱学校30所；试行以强带弱联盟发展 模式，加大教师交流力度，推进义务教育均衡发展，义务教育巩固率达到99.15%以上，白银区年内实现基本均衡，全市人口平均受教育年限提高到8.8年； 建设农村教师周转房100套、职业教育实训基地4个，新改扩建幼儿园25所；加快白银矿冶职业技术学院新校区建设步伐。推进县级公立医院改革，完善新农合 制度，巩固基本药物制度，实施中医药“3421”工程，建设15个乡镇卫生院，建成省中医院制剂中心，开工建设市第一人民医院住院部综合楼。深入创建全国 人口和计划生育利益导向政策体系示范区，稳定低生育水平，提高出生人口素质。办好省第二届中学生运动会，精心筹办省十三届运动会。

推进民生民利工作。健全社会保障体系，开展“社会养老服务体系建设推进年”活动，完善城乡低保对象动态管理机制，提高生活困难老人、未成年人、重度 残疾人、重病患者等重点救助对象社会救助水平，加强困难群体法律援助工作。改善城乡居住条件，为9200户发放廉租住房补贴。加强生活必需品市场价格监 测，推进放心粮油示范店建设，建立平价粮油、蔬菜直销点，确保群众基本生活稳定。做好全国第三次经济普查工作。

维护社会和谐稳定。全面开展“六五”普法，深入推进“法律七进”和法治城市创建活动，加快依法治市进程。落实安全生产“一岗双责”，建立安全生产隐 患排查治理体系，加大“打非治违”力度，杜绝重大事故发生。加强食品药品监管，启动食品药品检验所和检测技术评审中心建设。强化流动人口、特殊人群、“两 新”组织、互联网等领域社会管理创新,加快公共安全和治安防控体系建设，健全社会舆情分析研判、社会稳定风险评估和多元化纠纷解决机制。完善人民调解、司 法调解、行政调解相结合的大调解工作机制。开展双拥共建活动，巩固军政军民团结成果。

兴办八件惠民实事。（1）新建改建5家标准化县乡农贸市场；（2）建设150所农村互助老人幸福院；（3）建设白银城区公交候车亭220个；（4） 启动市儿童医院建设；（5）建设市青少年示范综合实践基地；（6）建成基层文化大院50家；（7）实施“五个一”全民健康教育工程；（8）实现乡镇、街道 政务服务中心和行政村、社区政务服务站点全覆盖。

加强政府自身建设

面对转型跨越的新形势、新任务，面对全市人民的新期待、新要求，我们将更充分地履行政府职能，更自觉地规范权力运行，努力建设人民满意的政府，开创政府工作新局面。

以敢为人先的气魄谋划发展。始终坚持解放思想，深化改革，以争先、领先、率先的气魄，创业、创新、创优的精神，开放、开明、开拓的理念，不断提高研 判形势、把握机遇、组织谋划和推动落实的能力。广泛听取社会各方意见，积极借鉴吸收成功经验，把优势运用到位，把潜力挖掘到位，把机遇转化为现实，把政策 转化为项目，以项目的大建设拉动大投资、培植大财源、带动大就业、促进大发展。

以攻坚克难的决心破解难题。以服务发展、服务企业、服务群众为出发点，深入开展下基层、进企业、联农户活动，从群众最关心、最直接、最现实的问题入 手，从企业发展最需要、最迫切、最突出的问题入手，正视经济结构单一、发展方式粗放的现实，直面发展基础不稳、质量不高、动力不足的矛盾，敢于担当负责， 敢于动真碰硬，敢于果断决策，紧扣项目建设、产业升级、富民增收、和谐稳定破解难题，不断完善政府决策机制，使政府的工作部署、政策措施更加符合实际，更 加顺应民意。

以优质高效的服务赢得先机。进一步清理、减少和调整行政审批事项，依法简化和规范审批程序，建立集中统一的公共资源交易监管服务平台，推进市县区政 务服务中心标准化、规范化建设，提高行政效能，改善发展环境。坚持依法行政，严格落实行政执法责任制。深入开展“效能风暴”行动，建立零距离服务、零关系 办事、零停留办公、零误差传递、零目标投诉，马上办、集中办、现场办、限时办“五零四办”工作制度，建设服务型政府，为各类市场主体提供更加优良便捷服 务，赢得发展先机。

以督查问责的机制狠抓落实。进一步充实完善目标管理内容，健全督查和通报机制，完善绩效考核评价和行政问责机制，科学合理确定评价标准，奖优罚劣， 确保各项重大决策、重要工作部署和目标任务的全面完成。探索运用企业标准化管理理念创新行政管理模式，在政府部门、事业单位推行ISO9000质量管理体 系试点，引进第三方评价，以管理创新推动工作落实，提高工作质量。

以廉洁务实的形象取信于民。自觉接受人大的法律监督和政协的民主监督，认真听取各民主党派、工商联、无党派人士的意见，支持工会、共青团、妇联等人 民团体更好地发挥作用，高度重视司法监督、舆论监督和社会公众监督。严格预算管理，强化税收征管，优化支出结构，把有限的资金更多地用在发展经济、改善民 生上。全面落实党风廉政建设责任制，健全完善廉政风险防控体系，强化行政监察和审计监督，严肃查处违法违纪案件。加大专项整治力度，坚决纠正工程建设、住 房保障、食品药品安全、环境保护等领域损害群众利益的不正之风，维护社会公平正义，以清正廉洁、勤俭节约、艰苦奋斗的本色凝聚力量，赢得民心。

各位代表！做好明年政府工作，任务艰巨，责任重大。我们要更加注重拓宽发展空间，更加注重提高发展质量，更加注重激发发展活力，更加注重共享发展成果。让 我们在中共白银市委的正确领导下，解放思想，务实创新，团结拼搏,苦干实干，为推进转型跨越发展、创造白银更加美好的明天而努力奋斗！
